# Alzheimer's Disease, Oestrogen and Mitochondria: an Ambiguous Relationship

**DOI:** 10.1007/s12035-012-8281-x

**Published:** 2012-06-08

**Authors:** Amandine Grimm, Yun-An Lim, Ayikoe Guy Mensah-Nyagan, Jürgen Götz, Anne Eckert

**Affiliations:** 1 Neurobiology Laboratory for Brain Ageing and Mental Health, Psychiatric University Clinics, University of Basel, CH-4012 Basel, Switzerland; 2 Equipe Steroïdes, Neuromodulateurs et Neuropathologies, Unité de Physiopathologie et Médecine Translationnelle, EA-4438, Université de Strasbourg, Faculté de Médecine, 67000 Strasbourg, France; 3 Centre for Ageing Dementia Research (CADR), Queensland Brain Institute (QBI), The University of Queensland, St Lucia Campus, Brisbane, QLD 4072 Australia

**Keywords:** Alzheimer's disease, Neurosteroids, Oestrogen, Mitochondria, ABAD

## Abstract

Hormonal deficit in post-menopausal women has been proposed to be one risk factor in Alzheimer's disease (AD) since two thirds of AD patients are women. However, large treatment trials showed negative effects of long-term treatment with oestrogens in older women. Thus, oestrogen treatment after menopause is still under debate, and several hypotheses trying to explain the failure in outcome are under discussion. Concurrently, it was shown that amyloid-beta (Aβ) peptide, the main constituent of senile plaques, as well as abnormally hyperphosphorylated tau protein, the main component of neurofibrillary tangles, can modulate the level of neurosteroids which notably represent neuroactive steroids synthetized within the nervous system, independently of peripheral endocrine glands. In this review, we summarize the role of neurosteroids especially that of oestrogen in AD and discuss their potentially neuroprotective effects with specific regard to the role of oestrogens on the maintenance and function of mitochondria, important organelles which are highly vulnerable to Aβ- and tau-induced toxicity. We also discuss the role of Aβ-binding alcohol dehydrogenase (ABAD), a mitochondrial enzyme able to bind Aβ peptide thereby modifying mitochondrial function as well as oestradiol levels suggesting possible modes of interaction between the three, and the potential therapeutic implication of inhibiting Aβ–ABAD interaction.

## Introduction

Steroid hormones are molecules, mainly produced by endocrine glands such as the adrenal gland, gonads and placenta, involved in the control of many physiological processes mainly in the periphery, from reproductive behaviour to stress response. In 1981, Baulieu and co-workers were the first to demonstrate steroid production within the nervous system itself [1]. They showed that the level of some steroids, such as dehydroepiandrosterone (DHEA), was even four times higher in the anterior brain of rats than in plasma and nearly 18 times higher than in the posterior brain with regard to its sulphated form (DHEAS). Of note, the level of this steroid remained elevated in the brain even after adrenalectomy and castration. In the following decades, other steroids were identified to be synthetized in situ in the brain, and enzymatic activities of proteins involved in steroidogenesis have been shown in many regions of the central and peripheral nervous system, in neurons as well as in glial cells [2–5]. Thus, this category of molecules is now called “neurosteroids” and defines neuroactive steroids that are synthetized within the nervous system, independently of peripheral endocrine glands. While steroid hormones act at a distance from their glands of origin in an endocrine way, neurosteroids are synthetized by the nervous system and act on the nervous system in an auto/paracrine configuration. Because of their lipophilic nature, peripheral steroid hormones can freely cross cell membranes, including the blood–brain barrier, and play an important role in the development, maturation and differentiation of the central and peripheral nervous system. However, since some steroids are also synthetized within the nervous system, their blood levels do not necessarily correspond to their brain concentrations [6]. Intra-cerebral steroid synthesis seems to play a role in cognition, anxiety, depression, neuroprotection and even nociception [7].

The ability to cross cellular membranes allows them to act on nuclear receptors exhibiting genomic action by regulating gene transcription. This action seems to be important during neonatal life where it has been shown that neurosteroids, such as progesterone (PROG) or oestradiol, are able to promote dendritic growth, spinogenesis, synaptogenesis and cell survival, particularly in the cerebellum [5]. Some studies already demonstrated the role of neurosteroids, particularly oestrogens, in the regulation of glucose homeostasis and lipid metabolism [8] as well as in neuroprotection [9]. Risk for Alzheimer's disease (AD) is associated with age-related loss of sex steroid hormones in both women and men [10, 11]. On the one hand, in post-menopausal women, the precipitous depletion of oestrogens and progestogens is hypothesized to increase susceptibility to AD pathogenesis, a concept largely supported by epidemiological evidence but refuted by some clinical findings, above all, by results from the “Woman's health initiative memory study” (WHIMS) (please see detailed discussion in the “Conclusion” section). On the other hand, a growing body of evidence indicates a more gradual age-related decline in testosterone in men similarly associated with increased risk to several diseases including AD. Since testosterone is at least in part aromatized in the brain to 17β-oestradiol, a loss of it may also affect oestrogen-mediated neuroprotective pathways. But also, the difference between how rapidly and significantly the female versus male primary sex hormones decline might partially contribute to higher AD incidences in women than in men [10].

## Alzheimer's Disease, Oxidative Stress, Effect of Gender and Neogenesis of Neurosteroids

AD is a neurodegenerative brain disorder and the most common form of dementia among the elderly as shown by the worldwide prevalence of the disease which was 26.6 million people in 2006 [12]. Clinical symptoms are characterized by severe and progressive loss of memory, language skills as well as spatial and temporal orientation. From a cellular point of view, the pathological hallmark of AD is the presence of extracellular senile plaques—composed of aggregated amyloid-β peptide (Aβ)—and intracellular neurofibrillary tangles (NFT)—consisting of aggregates of abnormally hyperphosphorylated tau protein. A lot of efforts have been made during the last years to understand the pathogenesis of the disease, particularly the role of AD key proteins, Aβ and tau, in oxidative stress and mitochondrial dysfunction [13].

Epidemiological and observational studies demonstrated a higher prevalence and incidence of AD in women even after adjusting for age—about two thirds of AD patients are female—as well as a greater vulnerability to the disease [14]. Thus, at early stages of neurofibrillary tangle development, women exhibit greater senile plaque deposition than men [15], and AD pathology is more strongly associated with clinical dementia in female patients than in male [16]. The drop of oestrogen levels after menopause was proposed to be one explanation to this phenomenon. However, there is little information concerning changes of steroid levels in the human brain during ageing and under dementia conditions. As steroids present in nervous tissues originate from the endocrine glands (steroid hormones) and from local synthesis (neurosteroids), changes in blood levels of steroids with age do not necessarily reflect changes in their brain levels. The concentrations of a range of neurosteroids have recently been measured in various brain regions of aged AD patients and aged non-demented controls including both genders by the very sensitive GC/MS methods [6]. Schumacher and colleagues showed a general trend towards lower level of steroids including oestrogen in AD patients compared to controls. Notably, neurosteroid levels were negatively correlated with Aβ and phospho-tau in some brain regions [6]. Another study using radioimmunoassay for steroid quantification demonstrated a decrease in oestrogen level in post-mortem brain from female AD patients aged 80 years and older but no significant difference in the 60–79-year age range compared to non-demented women [17]. However in men, an age-dependent decrease of androgen level was observed in the brain of non-demented subjects, which was even more pronounced in the brain of male AD patients [17]. Whereas large studies investigating systematically gender differences with respect to Aβ and or tau pathology in post-mortem brain tissue from AD patients are missing, broad evidence emerged from transgenic mice models of AD indicating an increased Aβ load burden and plaque number in the female brain compared to age-matched male mouse brain [11, 18]. Of note, consistent findings on greater Aβ burden in females were found in different animal AD models: Tg2576 (APPSWE) mice [19], APP/PS1 [20], APP23 [21], as well as in triple transgenic mice, like 3xTg-AD mice [18, 22] and ^triple^AD mice ([23], with respect to gender differences: unpublished observations). On the basis that the estrous cycle in female mice is constantly repeated until approximately 11 months of age and becomes irregular between 12 and 14 months, the data demonstrating a significant enhancement of Aβ load in important brain regions like the hippocampus from the female after the age of 11 months are striking. Regarding tau pathology, no gender differences have been observed in the latter triple AD models. In agreement, NFT formation in Aβ-injected tau transgenic mice (P301L) did not vary with gender [24]. Even though one single publication reported an enhanced neurofibrillary pathology in female TAPP mice [25], all together, these results point to the involvement of the Aβ pathway, rather than the tau pathway, in the higher risk of AD in women.

Interestingly, further supporting evidence comes from oxidative stress studies. Previous research of our group [26] demonstrated a gender-specific partial up-regulation of antioxidant defence in post-mortem brain regions from female compared to male AD patients further indicating that oxidative damage is caused rather by overproduction from reactive oxygen species (ROS) than by insufficient detoxification of ROS. Since mitochondria represent the major source of ROS, the findings from Lloret and co-workers are of specific interest showing that brain mitochondria from old female rats produce higher levels of ROS after exposure to Aβ than age-matched brain mitochondria from male rats [27].

A selection of studies attested neuroprotective effects of neurosteroids against AD-related cellular and mitochondrial injury, but the underlying mechanisms are still poorly understood.

**Fig. 1:**
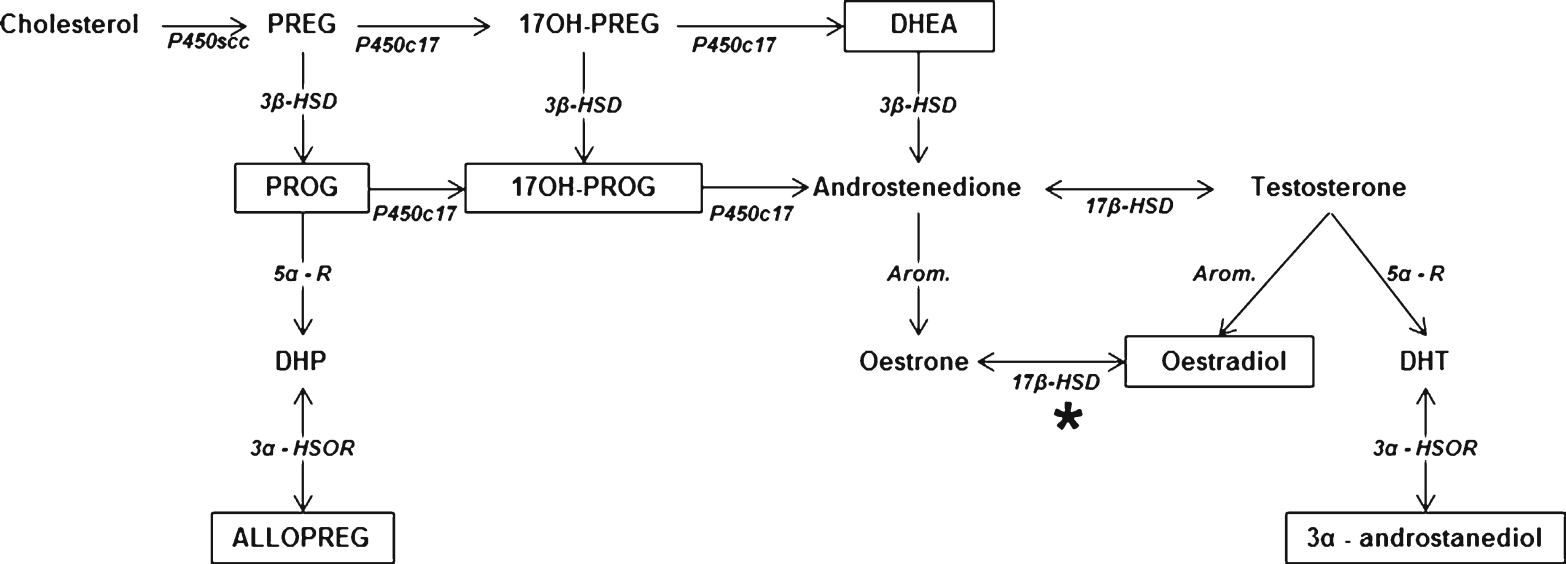
Main biochemical pathways for neurosteroidogenesis in the vertebrate brain. *Boxes* represent neurosteroids which are sensitive to modulation by AD key proteins, Aβ and/or tau. Mitochondrial 17β-HSD (marked by *) is equivalent to the ABAD in mitochondria. *PREG* pregnenolone, *PROG* progesterone, *17OH-PREG* 17-hydroxypregnenolone, *17OH-PROG* 17-hydroxyprogesterone, *DHEA* dehydroepiandrosterone, *DHP* dihydroprogesterone, *ALLOPREG* allopregnanolone, *DHT* dihydrotestosterone, *P450scc* cytochrome P450 cholesterol side chain cleavage, *P450c17* cytochrome P450c17, *3β-HSD* 3β-hydroxysteroid dehydrogenase, *5α-R* 5α-reductase, *Arom.* aromatase, *21-OHase* 21-hydroxylase, *3α-HSOR* 3α-hydroxysteroid oxydoreductase, *17β-HSD* 17β-hydroxysteroid dehydrogenase

Findings of our group corroborated that AD key proteins and oxidative stress are themselves able to modify neogenesis of neurosteroids in a cellular AD model [28, 29] (Fig. 1). In fact, treatment of human SH-SY5Y neuroblastoma cells with H_2_O_2_ for 24 or 48 h led to a decrease of oestradiol synthesis. This was paralleled by an increased cell death compared to untreated controls and a down-regulation of the expression of aromatase, an enzyme responsible for oestradiol formation from testosterone. Interestingly, cell death was also observed after inhibition of aromatase by treatment with letrozole, suggesting that endogenous oestradiol formation plays a critical role in cell survival. Furthermore, if cells were pre-treated with oestradiol, it was possible to protect them against H_2_O_2_ and letrozole-induced cell death. In agreement, a similar protective effect of oestradiol was observed in stress condition experiments treating the same cell line with heavy metals, such as cobalt and mercury [30].

In addition, modulation of neurosteroid production was observed in SH-SY5Y cells overexpressing the human amyloid processor protein (APP) or human tau protein [28]. Indeed, overexpression of human wild-type Tau (hTau 40) protein induced an increase in the production of PROG, 3α-androstanediol and 17-hydroxyprogesterone, in contrast to overexpression of the abnormally hyperphosphorylated tau bearing the P301L mutation which led to a decrease in the production of these neurosteroids. In parallel, a decrease of PROG and 17-hydroxyprogesterone production was observed in cells expressing human wild-type APP (wtAPP), whereas 3α-androstanediol and oestradiol levels were increased. These results provided first evidence that AD key proteins are able to modulate, directly or indirectly, the biological activity of the enzymatic machinery producing neurosteroids. These findings were further confirmed by in vitro experiments using native SH-SY5Y cells treated with aggregated Aβ_1-42_ peptide for 24 h [31]. Since APPwt SH-SY5Y cells secrete Aβ levels within nanomolar concentration range, treatment of native SH-SY5Y cells using a “non-toxic” concentration range (100–1,000 nM, non-cell death-inducing Aβ_1-42_ concentrations) revealed an increase in oestradiol production, whereas toxic Aβ_1–42_ concentrations within the micromolar range, leading to cell death, strongly reduced oestradiol levels.

Modulation of steroid production was also shown in other cell lines, for example in oligodendrocytes, where DHEA production is up-regulated under oxidative stress condition induced by treatment with Aβ peptide or Fe^2+^ [32]. Interestingly, similar results were found in Alzheimer patients where DHEA was significantly elevated in brain and cerebrospinal fluid when compared to control subjects [33]. Finally, several reports propose the role of allopregnanolone (3α, 5α-THP) as a plasmatic biomarker for AD, since it was shown that the level of this neurosteroid is decreased by 25 % in the plasma of demented patients compared with control subjects [34, 35].

The fact that the ability to produce neurosteroids is conserved in the vertebrates' evolution suggests that this category of molecules is important for living beings. Thus, we could speculate that the modulation of their biosynthesis plays an important role in the pathophysiology of neurodegenerative disorders, such as AD.

## Neurosteroids, Especially Oestrogens, and Neuroprotection in AD

### Evidence of Neuroprotective Action of Steroids in Cellular and Animal Studies

Neuroprotective effects of neurosteroids against a variety of brain injuries have already been described for many years. Numerous studies with the focus on oestrogens showed that these molecules are able to enhance cerebral blood flow, prevent atrophy of cholinergic neurons, and modulate the effects of trophic factors in the brain [36]. Oestrogens are a group of compounds known for their importance in the estrous cycle including oestrone (E1), oestradiol (E2), and oestriol (E3). Oestradiol is about ten times as potent as oestrone and about 80 times as potent as oestriol in its oestrogenic effect. Oestradiol is also present in males, being produced as an active metabolic product of testosterone. The serum levels of oestradiol in males (14–55 pg/mL) are roughly comparable to those of post-menopausal women (<35 pg/mL). Oestradiol in vivo is interconvertible with oestrone, oestradiol to oestrone conversion being favoured; however, evidence of metabolism is mainly derived from the periphery.

Animal studies, especially in rodents and transgenic mice models for AD, seem to confirm positive effects of oestrogen treatment. It has been shown that a treatment with oestrogen in mice expressing mutations in human APP (Swedish and Indiana mutation) had an impact on APP processing decreasing Aβ levels and so its aggregation into plaques [37]. Mechanisms underlying this action of oestrogen are still poorly understood, but as discussed by Pike et al. [11], it seems that oestrogen amongst others is able to promote the α-secretase pathway (non-amyloidogenic, meaning non-Aβ producing) via activation of extracellular-regulated kinase 1 and 2 (ERK 1 and 2) and through the protein kinase C (PKC) signalling pathway.

In triple transgenic AD mice, depletion in sex steroid hormones induced by ovariectomy in adult females increased significantly Aβ accumulation and had a negative impact on cognitive performance [18, 38]. Treatment of these ovariectomized mice with oestrogens was able to prevent these effects vice versa. Of note, when PROG was administrated in combination with oestrogens, the beneficial effects on Aβ accumulation were blocked but not on cognitive performance. However, oestrogen and PROG both can modulate kinase and phosphatase activity involved in tau phosphorylation, especially the glycogen synthase kinase-3β (GSK-3β). Thus, oestrogen can induce the phosphorylation of GSK-3β which inactivates the enzyme and reduces tau phosphorylation, whereas PROG can decrease the expression of tau and GSK-3β [11, 39]. This suggests that oestrogen and PROG not only can interact to regulate APP processing and tau phosphorylation but can also act independently on different AD pathways.

Cognitive effects of PROG were confirmed in mice bearing the Swedish double mutation of APP and mutant preseniline 1 (APPswe+PSEN1Δ9 mutant mice) which showed decreased hippocampally mediated cognitive performances compared to non-transgenic littermates [38]. In this AD mouse model, PROG was able to improve the cognitive performance in tasks involving the cortex but not in those involving the hippocampus. Besides, APPswe+PSEN1Δ9 mice presented decreased 3α, 5α-THP levels (metabolite of PROG) in the hippocampus, compared to wild-type mice, suggesting that deficits in hippocampal function may be due, at least in part, to reduced capacity to form 3α, 5α-THP in the hippocampus. Furthermore, a more recent study supported the role of 3α, 5α-THP in triple transgenic mice model of AD (3xTgAD) by showing reduced Aβ generation in the hippocampus, cortex and amygdala, coupled with an increased cellular regeneration after treatment with 3α, 5α-THP [40].

At the cellular level, oestrogen binds to nuclear receptors, such as oestrogen receptor α and β (ER α/β), and acts as transcription factor. It enhanced the expression of anti-apoptotic proteins, such as Bcl-2 and Bcl-xL, and down-regulated the expression of Bim, a pro-apoptotic factor, preventing the initialisation of cell death programme by mitochondria [11, 41]. Another way that oestrogen can protect cells from apoptosis is the activation of antioxidant defence systems by up-regulating the expression of manganese superoxide dismutase (MnSOD) and glutathione peroxidase [42]. Thus, oestrogen can have direct antioxidant effects by increasing reduced glutathione levels and decreasing oxidative DNA damage in mitochondria, as observed in a study using ovariectomized female rats [43]. Of note, oestrogen can also modulate the redox state of cells by intervening with several signalling pathways, such as mitogen-activated protein kinase (MAPK), G protein-regulated signalling, NFκB, c-fos, CREB, phosphatidylinositol-3-kinase, PKC and Ca^2+^ influx [41, 44]. On the basis of this complex mode of action, oestrogens not only seem to be able to decrease oxidative stress markers, including lipid peroxidation, protein oxidation and DNA damage, but can also directly act on the regulation of mitochondrial function [42].

### Neurosteroids and Mitochondria: Focus on Potential Protective Effects of Oestrogen Against Aβ-Induced Toxicity

Mitochondria are the “powerhouses of the cell”, providing the main part of cellular energy via ATP generation, which is accomplished through oxidative phosphorylation from nutritional sources [45]. They control cell survival and death by regulating both energy metabolism and apoptotic pathways and contribute to many cellular functions, including intracellular calcium homeostasis, alteration of the cellular reduction–oxidation potential, cell cycle regulation and synaptic plasticity [46]. Mitochondrial dysfunction has been proposed as an underlying mechanism in the early stages of AD [47, 48]. We recently summarized evidence from ageing and Alzheimer models showing that the harmful trio “ageing, Aβ and tau protein” triggers mitochondrial dysfunction through a number of pathways, such as impairment of oxidative phosphorylation, elevation of reactive oxygen species production and interaction with mitochondrial proteins, contributing to the development and progression of the disease [13, 49].

Mitochondria and neurosteroidogenesis are also closely linked since mitochondria contain the first enzyme involved in steroidogenesis, the cytochrome P450 cholesterol side chain cleavage enzyme (P450scc) located at the inner side of the mitochondrial membrane which is responsible for the conversion of cholesterol to pregnenolone (PREG). The first step of neurosteroidogenesis is the transfer of cholesterol from the outer to the inner mitochondrial membrane. It is also the rate-limiting step in the production of neurosteroids because the ability of cholesterol to enter into mitochondria to be available to the P450scc will determine the efficiency of steroidogenesis [50]. Free cholesterol accumulates outside of mitochondria and binds to the steroidogenic acute regulatory protein, a hormone-induced mitochondria-targeted protein that initiates cholesterol transfer into mitochondria. Then, molecules are transported inside mitochondria by a protein complex including translocator proteins (TSPO), a cholesterol-binding mitochondrial protein also known under the name of peripheral-type benzodiazepine receptor, which permits cholesterol transfer into mitochondria and subsequent steroid formation.

It has been shown that TSPO is up-regulated in the post-mortem brain of AD patients, resulting in an increased level of PREG in the hippocampal region of those brains [50]. Interestingly, the level of 22R-hydroxycholesterol, a steroid intermediate in the conversion of cholesterol to PREG, was found at lower levels in the AD brain compared to the control, which suggests that TSPO does not function normally in Alzheimer patients [33, 51].

**Fig. 2:**
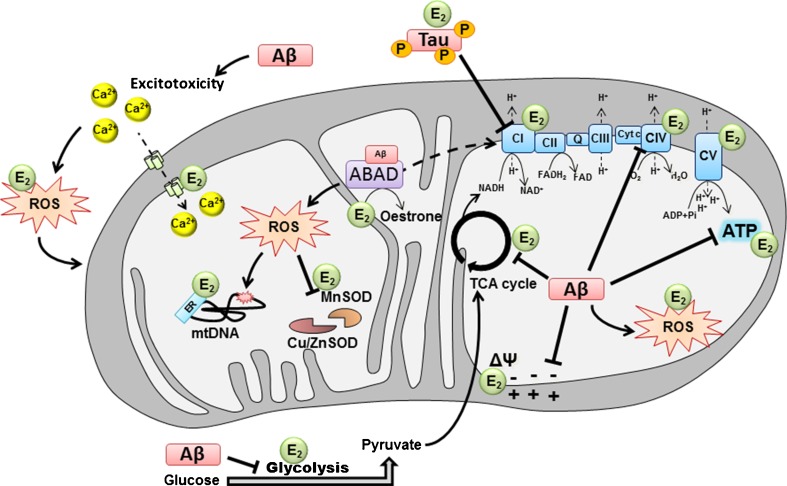
Modulation of mitochondrial function by Aβ, hyperphosphorylated tau and oestradiol. In AD, mitochondrial dysfunction was found to be a central pathological mechanism which occurs already at early stages of the disease. On one hand, studies showed that amyloid-β peptide (Aβ) can be responsible of metabolic impairments, such as the decrease of glucose consumption observed in the AD brain as well as the calcium-induced excitotoxicity in neurons. It has been found that hyperphosphorylated tau and Aβ are able to impair mitochondrial respiration by inhibiting the ETC CI and CIV, respectively, inducing decreased oxygen consumption, decreased ATP production and increased ROS level. This oxidative stress induced by ETC dysfunction can surpass cellular and mitochondrial scavenger (MnSOD, Cu/ZnSOD) and impacts on MMP as well as mitochondrial DNA (mtDNA). On the other hand, it has been shown that oestradiol can increase glucose utilization by cells as well as ETC activity, stabilize the MMP and prevent ROS production and calcium-induced excitotoxicity. In the graph, *E*_*2*_ designates where oestradiol potentially acts on mitochondria to compensate Aβ-induced toxicity. In turn, Aβ seems to be able to impact oestradiol metabolism in mitochondria, since it can be directly linked to the mitochondrial enzyme ABAD and possibly modulates its enzymatic activity (such as the reversible conversion of oestradiol to oestrone) and non-enzymatic activity (mitochondrial RNAse P). *ABAD* Aβ-binding alcohol dehydrogenase, *CI* complex I, *CII* complex II, *CIII* complex III, *CIV* complex IV, *CV* complex V, *cyt c* cytochrome c, *Cu/Zn SOD* copper/zinc superoxide dismutase, *MnSOD* manganese superoxide dismutase, *TCA* tricyclic acid, *E*_*2*_ oestradiol, *ROS* reactive oxygen species, *mtDNA* mitochondrial DNA, *ER* oestrogen receptor

From an energetic point of view, it is known that steroids such as oestrogen can regulate mitochondrial metabolism by increasing the expression of glucose transporter subunits and by regulating some enzymes involved in the tricarboxylic acid cycle (TCA cycle) as well as glycolysis, such as the hexokinase, phosphofructokinase, pyruvate and malate dehydrogenase [41, 52], which leads to improved glucose utilization by cells [11, 44] (Fig. 2).

Oestrogens seem to be able to up-regulate genes coding for some electron transport chain components present in nuclear and in mitochondrial DNA [53, 54]. In fact, an oestrogen-induced increased expression of some subunits of mitochondrial complex I (CI), cytochrome c oxidase (complex IV or CIV) and F1 subunit of ATP synthase was observed [41, 42, 52]. Furthermore, treatment of ovariectomized female rats with oestradiol induced an increase of mitochondrial respiratory function in the brain with regard to an enhancement of O_2_ consumption coupled to an increased activity of cytochrome c oxidase [53]. Thus, oestrogen seems to enhance the general metabolism in cells, but besides, it seems also able to directly protect mitochondria against oxidative stress-induced injury [52]. Thus, incubation of isolated mitochondria from the rat brain with oestradiol leads to a decrease of H_2_O_2_ production by this organelle coupled with an increase of the mitochondrial membrane potential (MMP). Furthermore, it has been proposed that its phenolic A ring could allow oestradiol to intercalate into the mitochondrial membrane and to avoid lipid peroxidation occurring in stress condition [54], which could be responsible for the stabilization of the MMP. Moreover, oestradiol seems to prevent the release of cytochrome c by mitochondria (a mechanism known to induce apoptosis of cells by activating the caspase cascade in the cytoplasm), a mechanism increasing the efficiency of the respiratory chain [52].

Finally, another oestrogen signalling pathway avoiding the negative effects of oxidative stress is the one regulating calcium homeostasis by inducing mitochondrial sequestration of cytosolic calcium [42, 54]. In fact, an imbalance of calcium regulation can lead to an increase of ROS production by activating the enzyme nitric oxide synthase, which can in turn sensitize neural cells to oxidative damage. It has been shown that an oestradiol treatment of primary hippocampal neurons was able to potentiate glutamatergic response via NMDA receptor which resulted in an increased influx of calcium in cells. This effect was coupled to an induction of mitochondrial sequestration of cytosolic calcium and an increase of the mitochondrial calcium load tolerability thereby avoiding calcium-induced excitotoxicity as well as promoting cell survival.

Taken together, all those different findings indicate that oestrogen might be able to compensate deficits and injuries that occur in AD, namely mitochondrial respiration impairments, enhanced ROS production, excitotoxicity and, more generally, metabolic deficits (Fig. 2). More recently, new light has been shed on a mitochondrial enzyme that is able to directly bind Aβ peptide and in which one of the main substrate is 17β-oestradiol [55]. This enzyme is known under the name of 17β-hydroxysteroid dehydrogenase type 10 (17β-HSD) or Aβ-binding alcohol dehydrogenase (ABAD).

### ABAD, Oestradiol and Aβ-Induced Mitochondrial Impairment

ABAD belongs to the alcohol dehydrogenase family, and it is responsible for the reversible oxido/reduction of several substrates including linear alcohols and steroids, such as 17β-oestradiol, using NAD^+^ as cofactor [56]. Under normal conditions (without Aβ), this enzyme plays a role in the regulation of metabolic homeostasis, and its overexpression improved cell viability and ATP content [57]. It has been shown that ABAD is up-regulated in brains of AD mice as well as AD patients [57, 58], and it has been suggested that the binding of Aβ changes the conformation of the enzyme, which seems to exacerbate mitochondrial dysfunction induced by Aβ.

More recently, studies performed in transgenic mice models of AD showed that behavioural stress or depletion of ovarian hormones by ovariectomy exacerbated mitochondrial dysfunction, aggravated plaque pathology and increased ABAD expression in the brain [59, 60]. Furthermore, double transgenic mice overexpressing mutant APP and ABAD present an earlier onset of cognitive impairment and histopathological changes when compared to APP mice [49], suggesting that Aβ–ABAD interaction is an important mechanism underlying Aβ toxicity. This hypothesis is supported by a study from Yao and collaborators who recently showed that inhibition of Aβ–ABAD interaction by a decoy peptide can restore mitochondrial deficits (activity of mitochondrial respiratory complexes, ROS level) and improve neuronal and cognitive function [60].

**Fig. 3:**
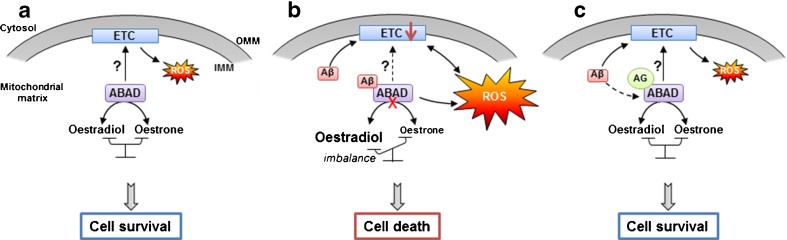
Aβ, ABAD and mitochondria: modes of interactions. **a** Under normal conditions, ABAD is responsible of the reversible oxido/reduction of linear alcohols and steroids, such as the reversible conversion from oestradiol to oestrone. Its potential function as an RNAse P could also be important for the good functioning of the mitochondrial ETC. **b** Under AD-relevant pathological conditions, Aβ can directly bind the mitochondrial enzyme ABAD, changing the configuration of the enzyme which seems to inhibit its activity and creates an imbalance between oestradiol and oestrone. Aβ-induced ABAD misfolding can impact ETC functioning and increase, directly or indirectly, ROS production, which lead to cell death. **c** In the presence of AG18051 (AG), the binding of Aβ to ABAD is inhibited, normalizing oestradiol level, ROS production, ETC activity, and improves cell survival. *ABAD* Aβ-binding alcohol dehydrogenase, *IMM* inner mitochondrial membrane, *OMM* outer mitochondrial membrane

New interesting findings of our group seem to go in the same way with regard to the use of a novel small ABAD-specific compound inhibitor (AG18051) by investigating the role of this enzyme in Aβ toxicity in human SH-SY5Y cells treated for 5 days with Aβ_1–42_ 0.5 uM [61]. The crystal structure of human ABAD in presence of AG18051 showed that the inhibitor formed a covalent link with the NAD^+^ cofactor and occupied the substrate-binding site of the enzyme [62]. Thus, the inhibitor was able to prevent Aβ-induced cell death and significantly normalized metabolic functions impaired by Aβ, such as cytosolic and mitochondrial ROS as well as mitochondrial respiration. Furthermore, it was able to restore oestradiol levels which were reduced after treatment with Aβ [31, 61]. What is interesting to note is that the apparent protective effects of the ABAD inhibitor seem to be independent on its interaction with Aβ. In fact, a 24-h pre-treatment with AG18051, before the incubation of cells with Aβ_1-42_, was sufficient to prevent cell death, normalize ROS production and restore mitochondrial respiration. Regarding oestradiol level, we previously showed that it decreased in the cytosol and increased in isolated mitochondria of SH-SY5Y cells after 5 days of treatment with Aβ [49]. The ABAD inhibitor normalized the oestradiol level in the cytosol [61], and preliminary data of our group suggest a similar effect in isolated mitochondria (unpublished data). Thus, we propose the following model of mode of action: ABAD inhibitor is able to block Aβ toxicity by changing ABAD configuration, which disables the binding of Aβ thus preventing its toxic effects (Fig. 3). The action of ABAD on the electron transport chain (ETC) is still unclear, but the potential role of ABAD as mitochondrial RNAse P directly links ABAD to the production of mitochondrial ETC proteins and ROS generation [63]. Notably, AG18051 was able to normalize also this function of ABAD since mitochondrial respiration was restored, but the underlying mechanisms still remain unclear [61].

Thus, the interplay between ABAD, oestradiol and mitochondria may be a very interesting lead to follow in the future to decode Aβ-induced mitochondrial toxicity and explore therapeutic strategies of ABAD inhibition.

## Conclusion

It is still debated whether oestrogen treatment after menopause could result in improved cognitive function in women. This debate is based on many animal and cell culture data showing that oestrogens can positively affect the ageing and AD brain. It was recognized from former studies that oestrogen depletion in post-menopausal women represents a significant risk factor for the development of AD and that an oestrogen replacement therapy may decrease this risk and even delay disease progression [64, 65]. However, large treatment trials showed negative effects of long-term treatment with oestrogens in older women. Above all, results from the WHIMS including 4,532 post-menopausal woman aged over 68 years indicated a twofold increase in dementia after 4.2 years of hormonal treatment (p.o. treatment with premaxin plus medroxyprogesterone). In addition, the study indicated potential risks for breast cancer, pulmonary embolism and stroke [66, 67]. Some attribute this failure to the synthetic nature of the hormones used in the WHIMS trial, since in vitro studies support a beneficial role of oestradiol and progesterone, but not of medroxyprogesterone used in the WHIMS [68, 69]. Of note, medroxyprogesterone is not metabolized to 3α, 5α-THP and can inhibit conversion of PROG to 3α, 5α-THP [70]. Similarly, oestradiol, PROG or 3α, 5α-THP, but not medroxyprogesterone, showed beneficial effects in ageing, seizure, cortical contusion, ischaemia and diabetic neuropathy models [38]. Another theory which tries to explain trial failure is the “critical window hypothesis”, asking for the critical period where oestrogen might exert a neuroprotective effect [71]. This hypothesis is substantiated by animal research, e.g. mice which have undergone ovariectomy, but in which oestrogen treatment was delayed substantially by months (the equivalent of years in human terms), did not benefit by this, as the animals did which received treatment immediately after ovariectomy [72]. However, a recent meta-analysis [73] indicated, contrary to expectations, that age of women and duration of time relapsed when treatment was initiated since menopause did not significantly affect treatment outcome. Thus, natural oestradiol (E2) without a progestagen should represent the preferred treatment [73]. Furthermore, the oral route of drug delivery, being non-invasive in nature, is by far the most convenient and preferred route of administration in any acute or chronic treatment. Though oestradiol itself from conventional oral oestradiol formulations has the ability to cross the blood–brain barrier (BBB) and reach the brain, but a large oral dose is required to achieve therapeutic levels of oestradiol due to its non-specificity for the brain. This non-specificity increases the peripheral drug burden and subsequently potentiates the risk of peripheral adverse effects. Furthermore, with specific regard to the brain-specific action of oestradiol as a neurosteroid, independently of its action in the periphery, other modes of administration (cyclical, nasal, polymer nanoparticles for oral delivery) need to be sought and investigated [74]. Alternatively, the true potential of phyto-oestrogens, like the soy isoflavones genistein, daidzein and glycitein, which activate the same neuroprotective pathways than oestrogens but with weak oestrogenic cellular effects that might be responsible for the lower prevalence of AD in Japanese living in their ethnic homeland compared to Japanese living in the USA [75], to beneficially modify disease processes should be studied in clinical trials [27]. In addition, the field could strongly benefit from the successful development of oestrogen derivates that have no unfavourable oestrogenic side effects. The successful use of oestrogen or oestrogen-analogue therapies to delay, prevent and/or treat AD will require additional research to optimize key parameters of therapy.

In this context, the interplay between ABAD, oestradiol and mitochondria and accordingly ABAD inhibition might represent a further interesting lead to follow in the future. Knowledge acquired from these studies will eventually be applied to unravel the pathophysiology and to inform prevention and intervention strategies of AD.
